# Investigating the Burden of Chronic Pain: An Inflammatory and Metabolic Composite

**DOI:** 10.1155/2016/7657329

**Published:** 2016-06-02

**Authors:** Kimberly T. Sibille, Ólöf A. Steingrímsdóttir, Roger B. Fillingim, Audun Stubhaug, Henrik Schirmer, Huaihou Chen, Bruce S. McEwen, Christopher S. Nielsen

**Affiliations:** ^1^Department of Aging & Geriatric Research, University of Florida, Gainesville, FL 32610, USA; ^2^Pain Research and Intervention Center of Excellence, University of Florida, Gainesville, FL 32610, USA; ^3^Department of Ageing and Health, Norwegian Institute of Public Health, 0403 Oslo, Norway; ^4^Department of Pain Management and Research, Oslo University Hospital, 0424 Oslo, Norway; ^5^Faculty of Medicine, University of Oslo, 0316 Oslo, Norway; ^6^Department of Clinical Medicine, Faculty of Health Sciences, The Arctic University, 9038 Tromsø, Norway; ^7^Department of Cardiology, University Hospital North Norway, 9038 Tromsø, Norway; ^8^Department of Biostatistics, University of Florida, Gainesville, FL 32610, USA; ^9^Harold and Margaret Milliken Hatch Laboratory of Neuroendocrinology, The Rockefeller University, New York, NY 10065, USA

## Abstract

*Background*. Chronic pain is associated with increased morbidity and mortality, predominated by cardiovascular disease and cancer. Investigating related risk factor measures may elucidate the biological burden of chronic pain.* Objectives*. We hypothesized that chronic pain severity would be positively associated with the risk factor composite.* Methods*. Data from 12,982 participants in the 6th Tromsø study were analyzed. Questionnaires included demographics, health behaviors, medical comorbidities, and chronic pain symptoms. The risk factor composite was comprised of body mass index, fibrinogen, C-reactive protein, and triglycerides. Chronic pain severity was characterized by frequency, intensity, time/duration, and total number of pain sites.* Results*. Individuals with chronic pain had a greater risk factor composite than individuals without chronic pain controlling for covariates and after excluding inflammation-related health conditions (*p* < 0.001). A significant “dose-response” relationship was demonstrated with pain severity (*p* < 0.001). In individuals with chronic pain, the risk factor composite varied by health behavior, exercise, lower levels and smoking, and higher levels.* Discussion*. The risk factor composite was higher in individuals with chronic pain, greater with increasing pain severity, and influenced by health behaviors.* Conclusions*. Identification of a biological composite sensitive to pain severity and adaptive/maladaptive behaviors would have significant clinical and research utility.

## 1. Introduction

Living with pain is linked with poor health and increased morbidity and mortality [1–5]. Individuals reporting “pain lasting at least one day during the past month” showed an increased incidence of and poorer survival rate from cancer [1] and an increased all-cause mortality over an eight-year period compared to those without pain [6]. Chronic widespread pain was associated with higher mortality rates over a 12-year period [3]. Further, Torrance and colleagues (2010) reported that severe chronic pain was related to increased all-cause mortality and to circulatory system disease deaths [5].

One method to initiate the investigation of the biological burden related to chronic pain is to explore biological measures associated with disease states linked with increased incidence of morbidity and mortality in individuals with chronic pain. For example, the leading causes of increased mortality reported in musculoskeletal and widespread pain conditions include cancer, cardiovascular disease, and other circulatory conditions [2, 3, 5, 7]. VanDenKerkhof and colleagues found that lifestyle factors (body mass index and smoking) and diet (in women) may contribute to the chronic widespread pain and cancer and cardiovascular disease relationship [8]. Additionally, cardiovascular risk factors related to metabolic syndrome were also associated with chronic pain [9]. Thus, investigating biological measures that are known risk factors for cardiovascular disease and cancer and that are associated with stress regulatory mechanisms, such as inflammatory and metabolic measures, may help elucidate the biological burden of chronic pain [10–12].

Frequently, research efforts have pursued the relationship of a specific disease condition by focusing on an isolated biological factor. However, biological measures do not work in isolation but rather interact in a complex array of systems [13, 14]. Additionally, the importance of considering a composite of measures reflecting system functioning has been well supported [15]. C-reactive protein (CRP), fibrinogen, body mass index (BMI), and triglycerides have been associated with the risk and/or progression of cancer and cardiovascular disease [16–18]. Importantly, in a large population based study [19], ten measures reflecting multiple biological systems were assessed and three measures were significantly associated with experiences of pain: BMI, serum triglycerides, and CRP. As previously noted, BMI was also associated with chronic wide spread pain in another large population-based study [8]. Additionally, fibrinogen, an inflammatory biomarker, was higher in individuals with versus without pain and fibrinogen levels predicted pain at 12 months [20].

The purpose of this study was to investigate if chronic pain is positively associated with a risk factor composite comprised of known inflammatory and metabolic measures linked with cancer and cardiovascular disease. We hypothesized that (1) individuals with chronic pain would demonstrate a higher risk factor composite compared to those individuals without chronic pain and that (2) a “dose-response” relationship would be demonstrated between chronic pain severity and the risk factor composite.

## 2. Methods

### 2.1. Sample

The Tromsø Study is a population-based study with participants recruited from the municipality of Tromsø in Northern Norway. The study thus far consists of six health surveys, carried out every six or seven years since 1974. All the surveys include questionnaire data, sampling of biological specimens, and clinical measurements. The different surveys are referred to as Tromsø 1–Tromsø 6 [21]. The current investigation is a cross-sectional design based data from the Tromsø 6 time point (collected from October 2007–December 2008). The main purpose of Tromsø 6 was to collect initial and repeated measurements of exposure data from new and previous participants of the Tromsø Study. The sample was recruited as follows: a 10% random sample of individuals aged 30 to 39 (*n* = 1,056), all residents aged 40 to 42 and 60 to 87 (*n* = 12,578), a 40% random sample of inhabitants aged 43 to 59 (*n* = 5,787), and all subjects who had attended the second visit of Tromsø 4 but not already included in the three groups above (*n* = 341). Of the invited 19,762 individuals, 65.7% participated in the Tromsø 6 survey (*n* = 12,981), 53.4% were women, and ages ranged from 30–87 [22].

### 2.2. Ethics

Tromsø 6 was approved by the Data Inspectorate of Norway and the present study was approved by the Regional Committee of Medical and Health Research Ethics, Northern Norway. The study complies with the Declaration of Helsinki, International Ethical Guidelines for Biomedical Research Involving Human Subjects, and the International Guidelines for Ethical Review of Epidemiological Studies. Participation was voluntary. Each subject gave written informed consent prior to participation in Tromsø 6 and for the subsequent scientific use of the data.

### 2.3. Questionnaires

All participants completed two questionnaires, Q1 and Q2. Q1, four pages in length, was distributed together with an invitation letter and completed before attending a medical examination. It covered topics such as demographics, education level, various health issues, symptoms, and diseases. Q2, 28 pages in length, covered more detailed information on topics already covered in the Q1, as well as some additional topics such as social network and life contentment. Q2 was completed either during the first visit or later at home and returned by mail. Those who answered in response to Q1 that they have had persistent or recurring pain with duration of three months or more were asked to fill in a more detailed pain questionnaire, as part of Q2. A list of the questionnaire items is available on the study homepage (http://tromsoundersokelsen.uit.no/tromso/).

### 2.4. Demographic Information

Participant age and sex were obtained from the Norwegian Central Population Registry. Information about education level was obtained from Q1. Analytically, age was treated as a categorical variable in the analysis of variance and as a continuous variable in the regression analysis. See Table 1 for age and education categorical definitions.

### 2.5. Health Behaviors and Mental Health

Data on smoking, alcohol consumption, and exercise were also obtained from the Q1. Health behavior categories implemented in analyses are listed in Table 1. Symptoms of anxiety and depression were assessed with the Hopkins Symptom Checklist- (HSCL-) 10. Participants scoring ≥1.85 were classified as having anxiety and depression [23].

### 2.6. Chronic Pain

Participants were classified as having chronic pain if they (a) answered “yes” to the question “Do you have persistent or recurring pain that has lasted three months or longer?” (b) stated that the pain occurred once a month or more frequently, and (c) rated average pain intensity as greater than zero on a 0–10-item numeric rating scale (NRS) with the anchors “no pain” and “the most intense pain imaginable.” Remaining participants were classified as not having chronic pain.

### 2.7. Chronic Pain Severity

Chronic pain severity was measured in the group of individuals who endorsed having chronic pain and completed a detailed pain questionnaire, as part of the Q2 which included the following questions; the response categories are listed in Table 2. The four domains were selected based on their relevance to chronic pain and their role in contributing to physiological change and biological system dysregulation [2, 5, 24–29]:

(i) Frequency:* How often do you have this pain?* (every day, ≥once a week, and ≥once a month).
(ii) Intensity:* How strong would you say that the pain usually is?* 10-item numeric rating scale:
 0 = no pain and 10 = worst imaginable pain.
(iii) Time (duration):* How long have you had this pain?* (years or months).
(iv) Total pain sites:* Where does it hurt?* (head/face, jaw/temporomandibular joint, neck, back, shoulder, arm/elbow, hand, hip, thigh/knee/leg, ankle/foot, chest/breast, stomach, genitalia/reproductive organs, skin, or other locations).

A 50% frequency split was performed for each dimension, dichotomizing the variable to a 0 or 1 score. Chronic pain severity, based on the four frequently captured pain dimensions, frequency, intensity, time (duration), and total number of pain sites (FITT), ranged from 1 to 5 designated by a cumulative score resulting from a combined total of the dichotomized values from the four pain dimensions [0 dimensions = a value of 1; one dimension = 2; two dimensions = 3; three dimensions = 4; and four dimensions = 5]. Pain dimensions are presented in Table 2.

### 2.8. Anthropometric and Biological Measures

Height and weight were measured on an automatic electronic scale [Jenix, DS 102 stadiometer (Dong Sahn Jenix, Seoul, Korea)]. Participants wore light clothing and no footwear. Body mass index (BMI) was calculated as weight in kilograms divided by the square of the height in meters (kg/m^2^).

The biological specimen collection and processing for Tromsø 6 are described in detail [22]. Nonfasting blood samples were collected twice, 50 mL at the first visit and 20 mL at the second visit. The participants were allowed to drink water and black coffee during their visits. Venipuncture was performed with subjects in a sitting position. A light tourniquet was used and released prior to the blood draw. After 30 minutes at room temperature, the coagulated samples were centrifuged at 2000 g for 10 minutes, and the sera were transferred within 1 hour to plastic tubes and kept between 1°C and 10°C. The blood samples were delivered twice daily to the Department of Laboratory Medicine, University Hospital Northern Norway, Tromsø, which is an accredited laboratory (ISO-standard 17025).

Fibrinogen was analyzed by a clotting assay with reagents from IL (Instrumentation Laboratory, Milano, Italy) on an ACL 3000 coagulation analyzer (Block Scientific Inc., Bohemia, New York). Serum triglycerides were analyzed within 10 hours by an enzymatic colorimetric method. CRP was analyzed by a highly sensitive CRP method (particle-enhanced immunoturbidimetric assay). Analyses were performed on a Modal PPE autoanalyzer with reagents from Roche Diagnostics Norway AS.

### 2.9. Risk Factor Composite

The selection of the four measures was based on prior findings [19, 20] and available biomarkers in the study: BMI, fibrinogen, CRP, and triglycerides. A combined biomarker composite was constructed, because (1) biological measures do not work in isolation but rather in conjunction with a complex array of measures [13, 29] and (2) a multisystem composite measure provides a stronger predictor over individualized or single system measures [15]. Due to skewed statistics, fibrinogen, CRP, and triglycerides were natural log transformed and then standardized to *z* scores, and normalization of the data was confirmed by review of histograms and summary statistics. BMI was standardized without transformation. The four *z* scores were then averaged to create a composite. Since minor deviations from normality in the component variables can produce deviation from normality in the risk factor composite, the final composite was *z*-transformed to ensure a mean of zero and SD of 1. A *z* score composite is an accepted formulation for biological measures allowing for the standardization of all measures regardless of different scales into the development of a continuous variable that is comparable across all participants in the study, particularly those with large sample sizes which allows for a strong population representation of the biological measures [30].

### 2.10. Statistical Analysis

Comparison of the risk composite in individuals with chronic pain versus those individuals without chronic pain was performed with a *t*-test. Multivariable comparisons were performed with analysis of variance (ANOVA), including sex, age group, education, health behavior variables (smoking, alcohol, and exercise), and mental health (HSCL ≥ 1.85) as cofactors. If nonsignificant (*p* > 0.05), the covariate was excluded. A sex × chronic pain interaction term was also tested in the model. Analyses were repeated excluding participants reporting a diagnosis of diabetes or attributing their pain symptoms to rheumatoid arthritis or ankylosing spondylitis in order to evaluate the relationship between chronic pain and the risk factor composite eliminating the influence of conditions associated with increased inflammatory and metabolic measures. Exclusion of the identified conditions also controls for medications associated with the treatment of those conditions, reducing the influence of specific medications on the risk factor composite [29].

The relationship between chronic pain severity and the risk factor composite was investigated in individuals with chronic pain with a stepwise multiple linear regression. In Step 1, chronic pain severity, based on a combined FITT measure, was entered along with covariates, sex, age group, education, health behavior variables (smoking, alcohol, and exercise), and mental health (HSCL ≥ 1.85), and then excluded if nonsignificant (*p* > 0.05). A sex × chronic pain severity interaction term was also included. Next, the health behavior variables were entered in Step 2. Mental health was entered in Step 3, that is, HSCL. If nonsignificant (*p* > 0.05), a covariate was excluded from the final model.

A post hoc analysis was performed on the individual pain dimensions to investigate their unique contribution. In Step 1 of the regression analysis pain duration, frequency, average pain intensity, and total number of pain sites were entered, along with sex, age, and education. Then health behaviors were entered in Step 2. Mental health, as measured by the HSCL, was excluded due to nonsignificance in prior models. Results were considered significant if *p* < 0.05 for all analyses. Lastly, a logistic regression was completed to determine the relative association of each biomarker to chronic pain.

## 3. Results

Participant demographics and covariates in the model are presented in Table 1. Additional information regarding Tromsø 6 sample representation is provided by Eggen and colleagues (2013). Distribution across the four pain dimensions, frequency, intensity, time (duration), and total pain sites and the chronic pain severity, based on combined FITT, are presented in Table 2. The mean and standard deviation for the individual components of the risk factor composite are BMI (26.9 ± 4.3), CRP (2.5 ± 4.6 mg/L), fibrinogen (3.5 ± 0.71 g/L), and triglycerides (1.5 ± 0.97 mmol/L). Following log transformations and standardization, the mean and standard deviation for the risk factor composite was 0 ± 1. A post hoc analysis was completed to determine the relative association of each biomarker to chronic pain; results are provided in Table 3.

### 3.1. Chronic Pain and the Risk Factor Composite

The risk factor composite was significantly higher among individuals with chronic pain (*M* = 0.13; SD = 0.98) compared to those without chronic pain (*M* = −0.07; SD = 0.98), *p* < 0.001. Multivariable ANOVA was performed, including sex, age, smoking, exercise, alcohol, and HSCL as cofactors in the analysis. The effects of chronic pain as well as the effects of all cofactors were significant in the analysis (*p* < 0.001), with the exception of HSCL which was not significant and excluded (*p* = 0.386); results are presented in Table 4. In addition, a significant sex by chronic pain interaction effect was found (*p* = 0.017). To explore this interaction the analysis was repeated for each sex independently. Chronic pain emerged as a significant predictor of increased risk factor composite for both sexes (*p* < 0.001), but the differences between individuals with chronic pain and the comparison group were greater among women than among men.

### 3.2. Comorbid Conditions

Additional analyses were completed excluding individuals reporting conditions that could contribute to elevated inflammatory and metabolic measures: diabetes (*N* = 634), rheumatoid arthritis (*N* = 261), and ankylosing spondylitis (*N* = 93). The overall sample size was thereby reduced by 958 participants; however the relationship between chronic pain and the risk factor composite, including the covariates described above, was essentially not affected (chronic pain, *β* = 0.17; *p* < 0.001).

### 3.3. Chronic Pain Severity

**Figure 1:**
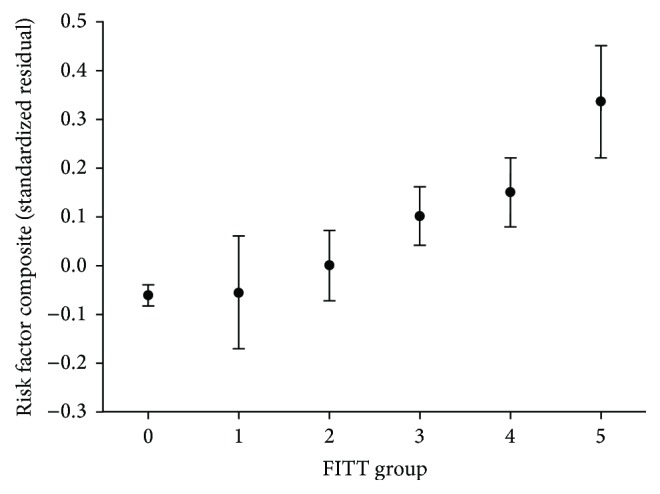
Risk factor composite by chronic pain severity groups. Adjusted for sex, age, education, alcohol use, exercise, and smoking. Chronic pain severity groups: pain dimensions: frequency, intensity, time, and total number of pain sites (FITT). 1 (low 4, high 0), 2 (low 3, high 1), 3 (low 2, high 2), 4 (low 1, high 3), 5 (low 0, high 4), and ^*∗*^0 added to reflect no chronic pain.

To explore whether there was a dose-dependent relationship between chronic pain severity and the risk factor composite, stepwise multiple linear regression analysis was performed on individuals who reported chronic pain, using a combined measure of pain frequency, intensity, time, and total number of pain sites (FITT) as the main predictor. In Step 1, age, sex, and education were included as covariates. Health behaviors (smoking, alcohol, and exercise) were added in Step 2, and HSCL was added in Step 3. As HSCL did not emerge as a significant predictor in the analysis, only results from Steps 1 and 2 are presented in Table 5. Male sex, increasing age, and previous and current smoking were all associated with a higher risk factor composite, whereas higher education, exercise, and alcohol consumption were found to be associated with a lower risk factor composite. After entering these variables, chronic pain severity (FITT) significantly predicted the risk factor composite (*β* = 0.11; *p* < 0.001, Step 2). Results are presented in Figure 1, where no chronic pain group is also included for comparison. A sex by FITT interaction was not statistically significant. However, a sex by alcohol use was significant, indicating that alcohol use was protective for women only. As described above, additional analyses were completed excluding individuals reporting conditions that could contribute to elevated inflammatory and metabolic measures; however, findings were minimally altered (FITT, *β* = 0.09; *p* < 0.001). The group difference in the mean risk factor composite between FITT group 1 and FITT group 5 is 0.40 (95% CI [0.24, 0.55]), which indicates a 0.4-standard deviation increase in the composite for those with severe chronic pain.

### 3.4. Chronic Pain Dimensions

**Figure 2:**
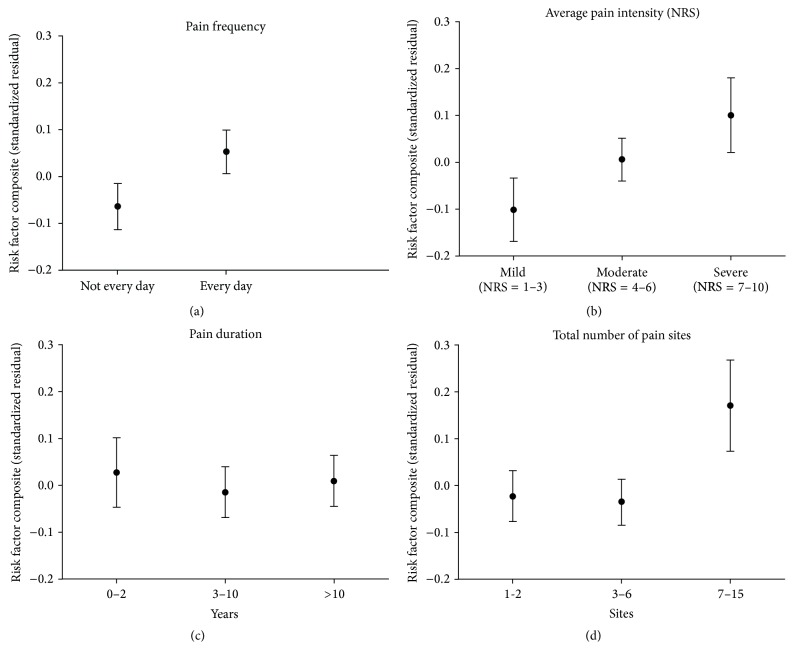
Risk factor composite by pain dimensions. Unique effect of each pain dimension after controlling for sex, age, education, exercise, alcohol consumption, smoking, and remaining pain dimensions.

A post hoc analysis was performed on all individuals reporting chronic pain to determine the contributions of the chronic pain severity measures: frequency, intensity, time (duration), and total pain sites. In the regression analysis all four variables were included along with all significant covariates from the main analysis for chronic pain severity. This analysis revealed that frequency (*β* = 0.57; *p* = 0.001), intensity (*β* = 0.74; *p* < 0.001), and total number of pain sites (*β* = 0.60; *p* = 0.001) were independently associated with the risk factor composite, whereas time (*β* = −0.02; *p* = 0.277) was not. Results are presented in Figure 2.

## 4. Discussion

Our findings are the first to link chronic pain to a composite of metabolic and immune measures associated with cardiovascular disease and cancer. We found that individuals with chronic pain have a higher risk factor composite than individuals without chronic pain. Second, we also for the first time demonstrated a “dose-response” pattern in the risk factor composite based on five levels of chronic pain severity even after controlling for relevant health behaviors and mental health symptoms and excluding inflammation and metabolic-related health conditions. Third, sex differences were highlighted across analyses providing validation of our findings. Lastly, health behaviors were associated with differing levels of the risk factor composite in individuals with chronic pain.

### 4.1. Chronic Pain

Our results provide evidence of a relationship between chronic pain and physiological system functioning, as previously proposed but not yet investigated [31–33]. Although the relationship between chronic pain and an array of individual biological measures has been investigated [34–36], we are the first to show the predictive utility of a composite measure reflecting immune and metabolic functioning. As biological measures do not function in isolation but rather contribute to dynamic, interactive systems, investigating a combination of measures reflecting overall system functioning has demonstrated greater predictive utility than evaluating individual measures [13–15, 29].

### 4.2. Chronic Pain Severity

Investigations of chronic pain are often limited to a dichotomous variable based on the report of pain experienced within a three- to sixth-month period which does not allow for differentiating from the longer term experiences and consequences associated with chronic pain. Differences in stress system responses have been reported in individuals with episodic pain compared to those with chronic pain following a low grade stressor [24]. Additionally, in individuals with a minimum duration of three years of episodic migraines, larger hippocampal volume and greater functional connectivity of the region were indicated in individuals with low frequency migraines compared to individuals with high frequency migraines [25]. This study is the first to stratify individuals with chronic pain by 5 levels of pain severity based on four common and frequently asked pain-related questions regarding frequency, intensity, time (duration), and total number of pain sites and demonstrate an increasing biological risk factor composite with increasing chronic pain severity.

In order to better reflect the biological burden of chronic pain, a quantifiable measure of an individual's pain experience is needed [10, 29]. For example, an individual with persistent, high intensity, chronic pain in multiple body sites, experienced for greater than eight years will likely have a different biological burden compared to an individual who has had an intermittent, low intensity, chronic pain for one year in one body site. Essentially, by categorizing chronic pain severity based on frequency, intensity, time (duration), and total number of pain sites, our findings support the above example: individual one's pain experience is consistent with pain severity FITT 5 and individual two with FITT 1. Of note, a medium effect size was indicated in the difference of the mean risk factor composite (0.4), from the least severe chronic pain group (FITT 1) to the most severe chronic pain group (FITT 5).

### 4.3. Pain Dimensions

Pain intensity, number of pain sites, and pain-related disability are associated with increased mortality [2, 5]. Our findings are specific to experiences of chronic pain and indicate that the individual dimensions of frequency, intensity, and total number of pain sites were significant predictors of the risk factor composite, while pain time (duration) was not. Results regarding duration may have been influenced by the high proportion of long-term chronicity of pain reported (median of 10 years). However, chronicity (duration) is relevant in combination with the severity of stress. As noted above, mild, intermittent, or occasional pain for years would not be expected to result in the same biological load when contrasted with persistent (daily), moderate-to-severe pain for years. Hence, it appears that the consideration of a combination of dimensions is important in developing an index that will characterize pain [26, 27, 37] as we demonstrate, to quantify the burden of chronic pain.

### 4.4. Sex Differences

An interaction between sex and chronic pain emerged such that differences in the risk factor composite between the no pain and chronic pain group were greater for women than for men. Although when analysis was limited to individuals with chronic pain, a sex and pain severity interaction was not indicated, male sex was associated with a higher risk factor composite. Sex differences in the prevalence and severity of chronic pain have been widely reported [38]. Particularly, in general, women show greater frequency of chronic pain and higher pain sensitivity. Less is known about sex differences in immune and metabolic measures [39], particularly in individuals with chronic pain. Consideration of sex differences in future investigations of stress-related biological measures and chronic pain is warranted.

### 4.5. Demographics and Health Behaviors

Increasing age and higher education were associated with lower and higher levels of the risk factor composite, respectively. Health behaviors such as exercise and alcohol consumption were associated with lower levels and smoking was associated with higher levels of the risk factor composite. A previous study demonstrated a relationship between previous lifestyle behaviors associated with increased risk for cancer and cardiovascular disease [body mass index, smoking, and diet (women)] and chronic widespread pain [8]. Importantly, the current study supports and extends the previously reported findings by showing that, in individuals with chronic pain, adaptive behaviors were associated with a lower risk factor composite and a maladaptive behavior was associated with a higher risk factor composite. Interestingly, retrospective behavioral self-reports are often limited by reporting bias and poor memory recall; however, each of the self-report indices demonstrated surprisingly predictive patterns in relation to the risk factor composite.

In regard to alcohol, health benefits have been reported with low-to-moderate intake, particularly in older women [40]. Greater than 16 units/month designation is within the low risk and moderate consumption guidelines [41]. Additionally, findings in a population-based study indicate that in individuals with chronic widespread pain the report of pain disability decreases with increasing consumption of alcohol until very high intake was reported, >35 units/week [42]. Most importantly, our findings suggest that (1) a biological composite may be sensitive to behavioral interventions and (2) adaptive and health promoting behaviors may reduce the biological load on the system and slow pathophysiological processes associated with chronic pain. These findings align with brain imaging findings indicating that adaptive behaviors modify the changes in the brain associated with chronic pain [37].

### 4.6. Theoretical and Mechanistic Considerations

There are a number of possible mechanisms contributing to the chronic pain and stress relationship. Dysregulated biological measures have been predictive of the onset of chronic pain and indicated in chronic pain conditions [20, 35]. Both acute pain and psychosocial stress can facilitate neuronal response amplification contributing to central nervous system pain sensitization, increasing the risk of chronic pain development [43–46]. Once pain becomes chronic, individuals not only experience the associated negative physiological arousal but also frequently report increased psychosocial distress [34, 47–50]. Additionally, health-related behaviors such as decreased exercise and physical activity, poor quality sleep, social isolation, and maladaptive coping patterns such as excessive alcohol consumption and smoking can further increase the physiological burden on an individual's system [51, 52].

Inflammation represents one potential mechanism linking chronic pain with increased morbidity and mortality [53, 54]. Inflammation is implicated in acute and chronic pain [36, 55, 56], is associated with psychosocial stress [57], and can be influenced by health behaviors such as exercise and sleep [58–60]. Similarly, dysregulated metabolic measures have been associated with morbidity and mortality [29, 30, 61]. The pathophysiological changes associated with chronic pain may contribute to dysregulated stress-related system functioning, a recognized pattern termed allostatic load [62]. Inflammatory and metabolic measures are included in studies of stress system functioning and allostatic load formulations [30]. Our findings of the dose response relationship between the chronic pain severity and the risk factor composite align with the allostatic load conceptualization.

### 4.7. Limitations and Future Directions

In order to fully evaluate the relationship of chronic pain on physiological functioning, additional biological measures need to be investigated including other inflammatory and metabolic indices as well as cardiovascular, neuroendocrine, and genetic [10, 62, 63]. Second, given our large sample size, a *z* score composite was implemented in this study due to its statistical advantages; however, a clinically derived risk factor composite has demonstrated utility and would be the next step for clinical investigation [30]. Third, the cross-sectional design limits causal inferences. Prospective studies will be an important next step to evaluate the relationships between changes in pain, comorbidities, health behaviors, and biological measures in order to better appreciate causal direction and possible modulating relationships. Regardless of causation, a composite measure of overall physiological functioning such as allostatic load, sensitive to biological, psychological, behavioral, and environmental changes, would be of great clinical and research benefit. Fourth, findings based on lengthy questionnaires can influence response rates. Importantly, the overall nonresponse rate for questionnaire completion was low with a 5–13% range indicated across the five primary pain questions. In general, a review of responses suggests that people tend to provide responses if they find the question meaningful. Fifth, further investigation of medication usage would be useful. We were not able to completely control for NSAID use, which would result in lower inflammatory levels, as such we are likely underestimating the relationship between chronic pain and the risk factor composite. Sixth, measuring chronic pain severity based on summing FITT domains is a new conceptualization and will require further testing, including comparing with other validated measures such as the Graded Chronic Pain Scale, and completing a formal validation study. Lastly, in the current investigation, the values used as cut-offs for dichotomizing each pain dimension were clinically quite high. The next step in this developing line of investigation would be to further explore optimal categorization of pain severity.

## 5. Conclusion

Individuals with chronic pain have a higher risk factor composite than individuals without chronic pain, and the risk factor composite increased in a dose-response fashion based on chronic pain severity (FITT), even after controlling for relevant cofactors and comorbidities. Our results build on previous findings regarding chronic pain-related increases in morbidity and mortality and support patterns identified in brain imaging studies indicating changes in structure and function are directly related to the frequency, intensity, and duration of pain and modified by health behaviors [37]. The identification and development of a biological composite measure sensitive to varying levels of chronic pain severity, adaptive/maladaptive behaviors, and pain treatment interventions would have significant clinical and research utility.

## Figures and Tables

**Table 1:** Participant representation by demographics and health behaviors.

** **	Women	Men	Both

Age group%			
30–44 years	22.9	21.2	22.1
45–59 years	27.6	28.7	28.1
60–69 years	30.4	33.0	31.6
70–87 years	19.1	17.1	18.2

Higher education%			
No	63.2	57.3	60.5
Yes	36.8	42.7	39.5

Chronic pain%			
No	59.4	71.3	65.0
Yes	40.6	28.7	35.0

Smoking%			
Never	40.4	33.8	37.3
Previously	38.2	46.9	42.3
Currently	21.4	19.3	20.4

Alcohol consumption%			
Never	14.7	7.8	11.5
<4 units per month	24.4	15.8	20.4
4–16 units per month	39.4	43.2	41.2
>16 units per month	21.5	33.2	27.0

Exercise frequency			
<Once per week	18.5	26.7	22.3
Once per week	18.5	21.4	19.9
2-3 times per week	40.7	36.3	38.7
>3 times per week	22.4	15.6	19.2

Note: higher education is defined as education > median for the age group.

**Table 2:** Pain dimensions: frequency, intensity, time, total pain sites, and chronic pain severity (FITT).

Pain dimensions	Category	Women	Men	Both
Frequency%	<Daily	44.5	45.4	44.8
*Dichotomous split *	Every day	55.5	54.6	55.2
*0 = <daily/1 = daily*				

Intensity% (NRS)	Mild pain (<4)	17.9	31.0	23.0
*Dichotomous split *	Moderate pain (4–6)	58.5	50.9	55.6
*0 = <5/1 = *≥*5*	Severe pain (7–10)	23.6	18.1	21.5

Time (duration)%	<1 year	6.5	8.1	7.1
*Dichotomous split *	1-2 years	11.8	14.1	12.7
*0 = <10 years*	3–5 years	19.6	21.7	20.4
*1 = >10 years*	6–10 years	21.0	20.8	20.9
	11–20 years	25.4	19.0	22.9
	>20 years	15.8	16.2	15.9

Total pain sites%	Single site	15.1	27.8	19.9
*Dichotomous split *	2-3 sites	30.8	41.2	34.8
*1–3 sites = 0*	4-5 sites	26.1	20.6	24.0
*4–15 sites = 1*	6–15 sites	27.9	10.4	21.3

FITT%	1 (4 low, 0 high)	6.4	11.7	8.5
*Combined splits*	2 (3 low, 1 high)	18.9	27.6	22.3
	3 (2 low, 2 high)	30.5	32.6	31.3
	4 (1 low, 3 high)	28.5	20.8	25.5
	5 (0 low, 4 high)	15.7	7.2	12.3

**Table 3:** Odds ratio of risk factors measures and relationship to chronic pain.

Odds ratio estimates				Pr > ChiSq
Biomarker	Point estimate^*∗*^	95% confidence interval		
		Lower bound	Upper bound	
ZLN-CRP	1.051	1.001	1.103	0.044
ZLN-FBR	1.102	1.052	1.154	<0.0001
ZLN-TRI	1.017	0.978	1.058	0.396
Z-BMI	1.137	1.091	1.184	<0.0001

ZLN-CRP: C-reactive protein (CRP) log transformed *z* score.

ZLN-FBR: fibrinogen log transformed *z* score.

ZLN-TRI: triglycerides log transformed *z* score.

Z-BMI: body mass index (BMI) *z* score.

^*∗*^A value greater than 1.0 is associated with increased odds for chronic pain.

**Table 4:** Multivariable ANOVA of chronic pain and the risk factor composite.

Parameter	β	Std. error	95% confidence interval		Sig.
			Lower bound	Upper bound	
Intercept	−0.051	0.039	−0.126	0.025	0.191
Sex	0.179	0.022	0.137	0.222	<0.001
AGEGRP3	0.517	0.03	0.459	0.575	<0.001
AGEGRP2	0.505	0.024	0.458	0.552	<0.001
AGEGRP1	0.302	0.024	0.255	0.349	<0.001
HIGH_EDU	−0.175	0.018	−0.211	−0.139	<0.001
SMOKING2	0.192	0.025	0.143	0.241	<0.001
SMOKING1	0.117	0.02	0.078	0.155	<0.001
ALCOHOL3	−0.336	0.033	−0.401	−0.27	<0.001
ALCOHOL2	−0.215	0.031	−0.276	−0.154	<0.001
ALCOHOL1	−0.125	0.033	−0.189	−0.06	<0.001
EXERCISE3	−0.529	0.028	−0.583	−0.475	<0.001
EXERCISE2	−0.309	0.024	−0.355	−0.263	<0.001
EXERCISE1	−0.148	0.027	−0.2	−0.095	<0.001
CPAIN	0.206	0.024	0.159	0.254	<0.001
Sex *∗* CPAIN	−0.087	0.037	−0.159	−0.015	0.017

Coding: dummy coding was completed for each of the following variables.

Age group (0 = 30–44; 1 = 45–59; 2 = 60–69; 3 = 70–87).

High education (0 = no; 1 = yes).

Smoking (0 = never; 1 = previously; 2 = currently).

Alcohol (0 = <2 units/mth; 1 = <3-4 units/mth; 2 = 5–16 units/mth; 3 = >16 units/mth).

Exercise (0 = <1x per week; 1 = 1x per week; 2 = 2-3x week; 3 = 4+x week).

**Table 5:** Predictive model of chronic pain severity (FITT) and the risk factor composite.

Model	*β*	Std. error	95% confidence interval		Sig.
			Lower bound	Upper bound	
(1) Constant	−1.034	0.102	−1.234	−0.833	<0.001
Sex	0.319	0.101	0.121	0.517	0.002
Age	0.013	0.001	0.01	0.016	<0.001
High_Edu	−0.222	0.037	−0.294	−0.15	<0.001
FITT_Total	0.126	0.02	0.087	0.165	<0.001
Sex_X_FITT	−0.055	0.032	−0.117	0.007	0.081

(2) Constant	−0.741	0.126	−0.987	−0.494	<0.001
Sex	0.287	0.099	0.092	0.481	0.004
Age	0.013	0.002	0.01	0.016	<0.001
High_Edu	−0.139	0.037	−0.211	−0.067	<0.001
FITT_Total	0.113	0.02	0.074	0.151	<0.001
Sex_X_FITT	−0.051	0.031	−0.111	0.01	0.099
Alcohol_1	−0.01	0.065	−0.139	0.118	0.875
Alcohol_2	−0.088	0.062	−0.21	0.034	0.156
Alcohol_3	−0.249	0.066	−0.378	−0.119	<0.001
Exercise_1	−0.147	0.052	−0.25	−0.044	0.005
Exercise_2	−0.286	0.046	−0.377	−0.195	<0.001
Exercise_3	−0.537	0.055	−0.644	−0.43	<0.001
Smoking_1	0.095	0.04	0.017	0.174	0.017
Smoking_2	0.168	0.047	0.075	0.26	<0.001

Coding: dummy coding was completed for each of the following variables.

Alcohol (0 = <2 units/mth; 1 = <3-4 units/mth; 2 = 5–16 units/mth; 3 = >16 units/mth).

Exercise (0 = <1x per week; 1 = 1x per week; 2 = 2-3x week; 3 = 4+x week).

Smoking (0 = never; 1 = previously; 2 = currently).
